# Management of overt upper gastrointestinal bleeding in a low resource setting: a real world report from Nigeria

**DOI:** 10.1186/s12876-014-0210-1

**Published:** 2014-12-10

**Authors:** Olusegun I Alatise, Adeniyi S Aderibigbe, Adewale O Adisa, Olusegun Adekanle, Augustine E Agbakwuru, Anthony O Arigbabu

**Affiliations:** Department of Surgery, College of Health Sciences, Obafemi Awolowo University Teaching Hospital Complex, PMB 5538, Ile-Ife, Osun State Nigeria; Department of Medicine, College of Health Sciences, Obafemi Awolowo University, Ile-Ife, Osun State Nigeria

**Keywords:** Upper gastrointestinal bleeding, Therapeutic, Surgery, Outcome, Nigeria

## Abstract

**Background:**

Upper gastrointestinal bleeding (UGIB) remains a common medical problem worldwide that has significant associated morbidity, mortality, and health care resource use. This study outlines the aetiology, clinical presentation, and treatment outcomes of patients with UGIB in a Nigerian low resource health facility.

**Methods:**

This was a descriptive study of consecutive patients who underwent upper gastrointestinal (GI) endoscopy for upper GI bleeding in the endoscopy unit of the Obafemi Awolowo University Teaching Hospital Complex, Ile-Ife, Osun State, Nigeria from January 2007 to December 2013.

**Results:**

During the study period, 287 (12.4%) of 2,320 patients who underwent upper GI endoscopies had UGIB. Of these, 206 (72.0%) patients were males and their ages ranged from 3 to 100 years with a median age of 49 years. The main clinical presentation included passage of melaena stool in 268 (93.4%) of individuals, 173 (60.3%) had haematemesis, 110 (38.3%) had haematochezia, and 161 (56.1%) were dizzy at presentation. Observed in 88 (30.6%) of UGIB patients, duodenal ulcer was the most common cause, followed by varices [52 (18.1%)] and gastritis [51 (17.1%)]. For variceal bleeding, 15 (28.8%) and 21 (40.4%) of patients had injection sclerotherapy and variceal band ligation, respectively. The overall rebleeding rate for endoscopic therapy for varices was 16.7%. For patients with ulcers, only 42 of 55 who had Forrest grade Ia to IIb ulcers were offered endoscopic therapy. Endoscopic therapy was áin 90.5% of the cases. No rebleeding followed endoscopic therapy for the ulcers. The obtained Rockall scores ranged from 2 to 10 and the median was 5.0. Of all patients, 92.7% had medium or high risk scores. An increase in Rockall score was significantly associated with length of hospital stay and mortality (p < 0.001). The overall mortality rate was 5.9% (17 patients).

**Conclusion:**

Endoscopic therapy for UGIB in a resource-poor setting such as Nigeria is feasible, significantly reduces morbidity and mortality, and is cost effective. Efforts should be made to improve the accessibility of these therapeutic procedure for patients with UGIB in Nigeria.

## Background

Upper gastrointestinal bleeding (UGIB) is a major affliction that has been known from antiquity and remains a common medical problem worldwide [1]. It is associated with significant morbidity, mortality, and health care resource utilization [2]. It is estimated that about 100–150 cases will occur per 100,000 adults per year in any given population [3,4]. About 4–14% of the affected patients will die from the condition, while 10–30% of patients will have rebleeding [5-7]. In resource-poor settings where facilities and the required equipment and training that are necessary for the management of this condition are limited, mortality will undoubtedly be higher.

Endoscopy has significantly revolutionised the management of patients with UGIB. It is used for diagnosis, stratifying patients, and treating the disease [6]. Therapeutic procedures, such as rubber band ligation, haemoclip, injection sclerotherapy and an endoscopic coagulation technique such as a heater probe, bipolar and monopolar coagulation, and argon plasma coagulation have been used to treat various causes of UGIB [6-8]. These treatment modalities have been shown to reduce rebleeding, blood transfusion requirements, and the need for ulcer surgery for definitive haemostasis [8]. In addition, appropriate use of endoscopy treatments significantly reduces hospital length of stay and health care costs [9-11].

In Nigeria and many economically resource-poor countries, upper gastrointestinal (GI) endoscopy services are not readily available. Where they are available, they are seldom affordable for most patients. This has limited the use of endoscopy in diagnosing the possible causes of bleeding. Worse still, the paucity of trained manpower in therapeutic procedures and the high cost of therapeutic endoscopic accessories have further limited the use of therapeutic techniques in the treatment of UGIB in Nigeria. The few centres that introduced therapeutic techniques had to adapt several modalities to reduce costs and maintain supplies [12]. Since the introduction of upper GI endoscopy to our hospital about three decades ago, few therapeutic procedures were performed, as reported in the literature [13]. Since 2007, different modalities of therapeutic procedures have been offered to patients that had overt UGIB. This study outlines the aetiology of UGIB, clinical presentation, treatment and outcomes of patients treated with endoscopy for UGIB. We identify the possible challenges preventing the routine use of these life-saving techniques in Nigeria.

## Methods

### Study design and setting

This was a descriptive study of consecutive patients who underwent upper GI endoscopy for UGIB in the endoscopy unit of the Obafemi Awolowo University Teaching Hospital Complex (OAUTHC), Ile-Ife, Osun State, Nigeria from January 2007 and December 2013. The endoscopy unit is open electively on weekdays and for emergencies on the weekend. OAUTHC is one of the first-generation teaching hospitals in Nigeria and it provides tertiary level services that are mostly accessed by patients from Osun, Ondo, Ekiti, and some parts of Kwara, Kogi, Oyo, and Edo states with an estimated population of about 10 million according to the 2006 national population census. The people of the Ife/Ijesa zone of Osun, where the institution is located, are predominantly Yorubas, who along with other Nigerian tribes reside permanently in this area. Peasant farming is the major occupation of these people. A sizeable number of people are engaged in commercial and small-scale industrial enterprises, whereas a significant number of the educated population are civil servants.

### Study subjects

The study subjects included all patients who had overt UGIB characterised by passage of melaena stool, haematemesis and/or haematochezia, whose sources of the bleeding were identified from the oesophagus, stomach or duodenum at upper gastrointestinal tract endoscopy and who signed informed consent. When multiple sources were identified, the principal cause of bleeding was ascertained based on the clinical presentation and endoscopic findings. All patients who had a bleeding episode from other causes during hospitalisation were excluded from the study. Ethical clearance was obtained before the commencement of this study from the Obafemi Awolowo University Teaching Hospitals Complex Ethics and Research Committee.

### Procedures

Most of the patients were admitted through the emergency department of the hospital. Generally, patients were resuscitated with intravenous fluids, blood and blood products as indicated. A nasogastric tube was inserted to monitor continuous bleeding. Endoscopy was performed within 48 hours of admission especially when there was evidence of continuous bleeding, features of haemorrhagic shock at presentation and patients having funds for the procedure. Those patients that were referred from other hospitals after resuscitation had the procedure done at the next available endoscopy session. Proton pump inhibitors were used routinely for patients with suspected peptic ulcer bleeding at presentation while beta blockers were administered routinely to patients with variceal bleeding once they were stabilised. Somatostatin analogues and interventional radiology facilities were not available and were therefore not used in any of the patients. The therapeutic methods used included injection sclerotherapy, rubber band ligation, heater probes and bipolar coagulation. Patients with variceal bleeding received injection sclerotherapy or rubber band ligation. The method of therapy offered was also determined by what patients can afford. Patients who had non-variceal bleeding, especially with gastric and duodenal ulcers, received a combination of therapy that included sclerotherapy and thermal therapy. Only ulcer patients who had a Forrest Grade 1a to IIb ulcers were routinely scheduled for endoscopic therapy. Agents used for the injection therapy included adrenalin and/or 50% dextrose water. Patients with failed endoscopy therapy were offered surgery.

### Data collection

Data obtained included sociodemographic data, cause of disease, clinical presentation, blood investigations including complete blood count and clotting profile, endoscopic findings, treatment modalities, and clinical outcome (i.e., transfusion requirements, length of hospital stay, rebleeding rate, and mortality). Endoscopic evaluation of the bleeding lesion in the case of peptic ulcer was defined according to the Forrest classification system as follows: FI (FIa and FIb), FII (FIIa, FIIb and FIIc), and FIII [14]. Patients who had a variceal type of upper GI bleeding were classified endoscopically according to the severity of varices into four grades (i.e., grades I–IV) [15]. The grade assigned to each of the patients was based on the highest grade observed in each patient. Gastric extension of the oesophageal varices were classified using Sarin’s classification into GOV-1 or GOV-2, while isolated gastric varices were classified as IGV-1 and IGV-2. Endoscopic diagnosis was considered to be accurate if stigmata of active or recent bleeding were present, independently of the nature of the bleeding lesion. Normal examination was defined by the absence of any endoscopic abnormality. Shock was defined as a systolic blood pressure below 90 mmHg at presentation. Rebleeding was defined as a new bleeding episode from the same source within 6 months after the initial bleeding had stopped. The post-endoscopy Rockall risk scoring system, which uses clinical (e.g., age, co-morbidity, and presence of shock) and endoscopic (e.g., diagnosis of stigmata of recent haemorrhage) criteria, was used to predict patients at risk for developing adverse outcomes after acute UGIB [16].

### Statistical analyses

Data analysis was performed using the SPSS statistical package for social sciences (SPSS, version 21.0; Chicago, IL, USA) program. The mean ± standard deviation (SD), median and ranges were calculated for continuous variables, whereas proportions and frequency tables were used to summarise categorical variables. Continuous variables were also categorised. A Chi-square (χ^2^) test was used to test for the significance of association between the independent (predictor) and dependent (outcome) variables for categorical variables. Correlation analysis was used to assess the relationship between Rockall scores and the number of blood units transfused and durations of hospital stay. The level of significance was considered as P < 0.05.

## Results

### Demographic characteristics

**Figure 1:**
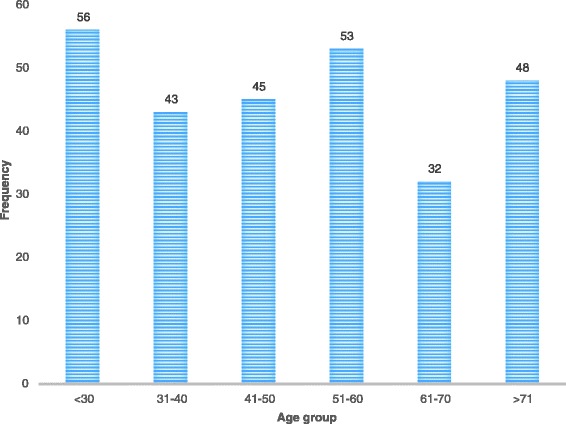
**Age (years) distribution of patients with upper gastrointestinal bleeding.**

During the study period, a total of 2,320 patients underwent upper GI endoscopy, of which 287 (12.4%) had UGIB. Two hundred and six (72.0%) patients were males while 80 (28.0%) were females, with a male to female ratio of 2.6:1. Patient ages ranged from 3 to 100 years with a median age of 49 years. As shown in Figure 1, different age groups were affected almost equally.

The patients had varying levels of education. Thirty-nine (13.6%) patients had no formal education, 58 (20.2%) had only a primary level education, 67 (23.4%) had a secondary level education and 123 (42.8%) had a tertiary level education. The patients were from different walks of life. Sixty-one (21.3%) of the patients were traders, 54 (18.8%) were civil servants, 41 (14.3%) were students, 34 (11.9%) were self-employed or artisans, 33 (11.5%) were retired, 28 (9.8%) were farmers, 20 (7.0%) were clergy, and 16 (5.6%) were unemployed.

### Clinical characteristics of patients

Passage of melaena stool was the most common [268 (93.4%)] presenting complaint. One hundred and seventy-three (60.3%) and 110 (38.3%) patients presented with haematemesis and haematochezia, respectively. Slightly over half of the patients [161 (56.1%)] were dizzy at presentation. Similarly, 152 (53.0%) presented with abdominal pain, 27 (9.4%) presented with jaundice and 113 (39.4%) had a history of recent ingestion of non-steroidal anti-inflammatory drugs.

The use of alcohol or tobacco was reported in 35 (12.1%) and 8 (2.8%) patients, respectively. Previous history of dyspepsia was noted in 123 (42.9%) patients. Comorbidities included hypertension in 68 (23.7%), chronic liver disease in 45 (15.7%), diabetic mellitus in 11 (3.8%), previous cerebrovascular disease in 6 (0.7%) and renal impairment in 4 (1.4%) patients. Eighty-nine (31.0%) patients presented with haemorrhagic shock.

### Endoscopic diagnosis

As shown in Table 1, the sources of bleeding were endoscopically identified in 265 (92.3%) patients. Duodenal ulcer [88 (30.6%)] was the most common cause of UGIB, followed by varices [52 (18.1%)] and gastritis [51 (17.1%)]. Among patients with variceal bleeding, all had oesophageal varices with gastric extension. According to Sarin’s classification, GOV-1 was found in 41 (78.8%) patients while GOV-2 was seen in 11 (21.2%) patients. Four (7.8%) of these patients also had associated isolated gastric varices. Of these, three were IGV-1 and one was IGV-2. All patients had at least four oesophageal variceal columns. No patient had grade I, seven (13.4%) patients had grade II, thirty-seven (71.2%) patients had grade III, and eight (15.4%) patients had grade IV.

**Table 1:** **Spectrum of aetiological findings at upper GI endoscopy in patients with upper gastrointestinal bleeding**

**Endoscopic diagnosis**	**Frequency**	**Percentage**
Duodenal Ulcer	88	30.6
Varices	52	18.1
Gastritis	51	17.1
Gastric ulcer	30	10.4
Normal finding	22	7.6
Gastric cancer	21	7.3
Reflux esophagitis	8	2.8
Gastrointestinal stroma tumour	8	2.8
Gastric polyp	4	1.4
Vascular bleeding	1	0.4
Mallory Weiss tear	1	0.4
Oesophageal cancer	1	0.4
**Total**	287	100.0

According to Forrest classifications, the endoscopic grade for gastric and duodenal ulcers were as follows; Grade Ia: 3 (2.5%) patients, Grade Ib: 15 (12.7%), Grade IIa: 16 (13.6%), Grade IIb: 21 (17.8%), Grade IIc: 36 (30.5%) and Grade III: 27 (22.9%).

### Admission and treatment patterns

A total of 254 (88.5%) patients were admitted for the management of upper GI bleeding either in our hospital or the referring hospital. The treatments offered are shown in Table 2. Of the 52 patients with variceal bleeding, 15 (28.8%) had injection sclerotherapy to stop active bleeding. Five (33.3%) patients had rebleeding from the varices after injection therapy. Twenty one (40.4%) patients had variceal band ligation. Of these, 18 patients had two sessions for variceal eradication. Rebleeding from the varices occurred in one patient who had one session of variceal ligation within 2 weeks of the procedure. Sixteen (30.8%) patients who did not consent or could not afford the cost of the therapeutic procedure were untreated. Twelve of the sixteen had recurrent bleeding episodes and eventually died of the bleeding within one year of follow up. The overall rebleeding rate in patients that had endoscopic therapy was 16.7% as compared to 75.0% of patients who did not have any form of endoscopic therapy, and this was a statistically significant difference (p < 0.001).

**Table 2:** **Treatment pattern among patients with upper GI bleeding**

**Treatment pattern**	**Number of patients**	**Percentage**
Medical (conservative) treatment	186	64.8
Endoscopic treatment	82	28.6
Surgical treatment	19	6.6
Total	287	100

For patients with ulcers, of the 55 patients who were eligible for endoscopy therapy, only 42 patients could afford endoscopic treatments. Endoscopic therapy was effective in 90.5% of the cases, while control of bleeding could not be achieved in four (9.5%) patients. Of the 4 patients that died, 3 had surgical procedures and one died before surgical intervention could be offered. One patient who had surgical haemostasis rebled 2 months after the procedure and died before arriving at the hospital. No rebleeding was observed in patients that had endoscopic haemostasis during the period of follow up. Of the 13 patients who did not have any endoscopic therapy, five (38.5%) had rebleeding necessitating readmission. All the five but two patients benefited from endoscopic therapy, and surgical haemostasis was achieved in the two patients who did not receive endoscopic therapy. All patients with gastritis were treated with medical therapy. No recurrence was recorded in any of these patients.

Table 3 shows the indication for surgery in patients that had UGIB in our study. Six patients who had endoscopic features of gastric cancer had active bleeding at endoscopy. They had emergency surgery to control the bleeding. Three patients each had surgical resection as well as gastrotomy and haemostasis with electrocoagulation. Surgical resections were also offered to patients who had gastrointestinal stroma tumours (GIST). Endoscopic polypectomy was offered to patients who had bleeding gastric polyps. Table 4 shows the procedures performed in 19 patients that had surgical interventions.

**Table 3:** **Indications for surgery in patients with upper gastrointestinal bleeding (n = 19)**

**Indication for surgery**	**Frequency**	**Percentage**
Bleeding duodenal ulcers not responding to either medical or endoscopic treatment	5	26.3
Gastric malignancy	6	31.6
Gastrointestinal stroma tumour	8	42.1

**Table 4:** **Types of surgery performed in patients with upper gastrointestinal bleeding**

**Type of surgery**	**Frequency**	**Percentage**
Under-running of bleeding vessel	5	26.3
Gastrostomy	3	15.8
Subtotal gastrectomy	3	15.8
Partial gastrectomy	8	42.1

### Rockall scoring system

The Rockall score was calculated for all patients. The scores obtained ranged from 2 to 10 and the median was 5.0. Of these, 21 (7.3%) patients had Rockall scores <3 (low risk group), 253 (88.2%) had scores of 3–8 (medium risk group), and 13 (4.5%) had scores >8 (high risk group). A higher Rockall score was significantly associated with length of hospital stay and mortality (p < 0.001).

### Blood transfusion requirement

The median admission pack cell volume was 20.5% and the range was 9–35%. Two-hundred-and-forty (83.6%) patients had a blood transfusion during the course of their treatment. The number of 500-ml units of blood transfused ranged from 1 to 32. The median number of transfusion units was three. The blood transfusion units given were significantly different in patients who had endoscopy therapy compared to the non-endoscopy therapy group in patients with variceal and duodenal bleeding (p = 0.031 and p = 0.01, respectively; Table 5). However, the transfusion requirements were not significantly different in patients who survived versus those who did not (p = 0.089), as well as in relation to Rockall scores (p = 0.484).

**Table 5:** **Mortality, rebleeding, blood transfusion requirement, and length of hospital stay for endoscopic and non-endoscopic therapy**

	**Endoscopic therapy**	**Non endoscopic therapy**	**p**
Varices	36	16	
Mortality in six months	2	8	<0.001
Rebleeding	5	12	<0.001
Blood transfusion units Mean ± SD	3.0 ± 3.6	4.7 ± 5.7	0.031
Hospital stay (in days) Mean ± SD	5.0 ± 1.6	9.2 ± 5.4	<0.001
Duodenal ulcer	42	13	
Rebleeding	0	5	<0.001
Blood transfusion units Mean ± SD	2.8 ± 3.4	3.1 ± 3.1	0.01
Hospital stay (in days) Mean ± SD	3.7 ± 2.0	9.4 ± 4.9	<0.001

### Length of hospital stay

The overall length of hospital stay ranged from 1 to 20 days with a median of 4.5 days. Length of hospital stay was significantly shorter in patients who received endoscopy therapy for variceal and ulcer bleeding when compared with those who did not receive therapy (p < 0.001; Table 5). The length of hospital stay was shorter after endoscopic therapy when compared with those who had surgical intervention (P < 0.001).

### Mortality

The overall mortality rate was 5.9% (17 deaths). Of these, 13 (67.4%) patients had variceal bleeding. Mortality was significantly higher in those patients who did not have any form of endoscopic therapy compared with those who were offered this therapy (p < 0.001; Table 5). Two (11.8%) patients died following treatment for ulcers. Two (11.8%) patients who had gastric resection for cancer died during the perioperative period.

## Discussion

Upper gastrointestinal bleeding continues to be a significant cause of morbidity and mortality worldwide, which places a significant demand on healthcare facilities [2-4,17]. In our experience, it is one of the leading causes of referral for upper GI endoscopy. We found that 12.6% of the endoscopy referrals were for UGIB. This is higher than previously reported from our centre, [18] but similar to findings reported by Malu et al. [19] in their series from Northern Nigeria. However, Olokooba et al. [20] reported a high rate in the middle belt of Nigeria. This disparity may be due to the fact that the latter was an initial report with a small sample size. Being an initial report, the referrals were likely those that would benefit from the upper GI endoscopy the most.

Upper GI endoscopy remains the most effective modality to localise the site and cause of bleeding in over 95% of patients, especially when the procedure is performed within 24 hours of bleeding [5,21,22]. In the present study, the site of bleeding was detected in 91.6% of patients, which was similar to findings previously reported [23,24]. Not all patients had the endoscopy procedure done within 48 hours of the initial occurrence of bleeding. Mucosal lesions are well known to heal quickly and generally the time interval between the bleeding episode and the endoscopic procedure is known to influence the accuracy and likelihood of finding a cause endoscopically [25]. Therefore, the availability of emergency endoscopy within 24 hours is most desirable. In reality, however, emergency endoscopy is rarely available in most healthcare centres in developing countries due to the insufficiency of well-trained endoscopists, trained support staff, or equipment. In addition, difficulty in accessing tertiary health services due to the poor referral systems that characterise most developing countries also prevents early presentation and detection of the cause of bleeding.

Our finding that duodenal ulcers were the most common cause of upper gastrointestinal bleeding in our study is consistent with reports from most Western countries [26-28]. Most studies from the Western world report that almost half of their cases with UGIB are due to duodenal ulcers, which is higher than the finding in this study [26-30]. The possible reason may be because of the fact that the median age of our patients was almost a decade lower than the average age in most Western case series [28,29]. It is worth noting that variceal bleeding was the most common cause of UGIB in Northern Nigeria and most parts of North Africa where there is a high prevalence of chronic liver disease due to hepatitis B infection and schistosomiasis [19,22,25,31-33]. This implies that the most common cause of UGIB in any given environment may be influenced by the prevalence of hepatitis B virus and schistosomiasis. However, we found that oesophageal varices were the second-most common cause of UGIB in our cohort. All of the patients in our study had a history of liver disease from hepatitis B or C virus. None of the patients had screening or preventive therapy for cirrhosis. In fact the diagnosis of cirrhosis was heralded by the occurrence of variceal bleeding in some of these patients. This implies that public education must be undertaken to inform people of the early features of liver disease and the advantages of early diagnosis.

Furthermore, in our study, gastritis accounted for the third-most common cause of UGIB. It should be noted that Ajayi et al. reported that gastritis was the most common cause of UGIB in their case study from southwest Nigeria [34]. Most of our patients were either farmers, artisans or traders. This group of patients is known to work long hours and they often abuse over-the-counter analgesics including nonsteroidal anti-inflammatory drugs (NSAIDs). Hence, gastritis and peptic ulcer-related causes were a common cause of UGIB in our series. This proposition is supported by the fact that almost 40% of our patients gave a history of recent or chronic use of NSAIDs. This finding was similar to a previous report from Nigeria [20]. However, the percentage is low compared to another study from Africa [22]. Data from Spain indicated that more than 50,000 gastrointestinal bleeding events and more than 1,000 deaths yearly were attributable to aspirin or other NSAID use [35,36]. This national statistic translates to an NSAID-associated mortality of 5.6% or 15.3 deaths per 100,000 users [36]. Mortality from NSAIDs has, however, been reduced significantly because of a decrease in the prevalence of *Helicobacter pylori* infection as a result of frequent use of over-the-counter antibiotics. Additionally, increased use of proton pump inhibitors, widespread efforts to use lower doses of NSAIDs, increased use of safer NSAIDs, and improved treatment of acute ulcer bleeding have also contributed to lower mortality rates [37-40]. However, in Nigeria where there is a high prevalence of *H. pylori* [41] and indiscriminate use and purchase of NSAIDs across the counter, the morbidity and mortality of NSAID use may actually be on the rise. Regulations to discourage the prescription and dispensing of drugs by unqualified personnel are of paramount important in reducing this negative trend.

A previous report demonstrated that approximately 80% of patients with UGIB will experience an end of bleeding especially after treatment with medical therapy [30]. In this index study, however, only about two-thirds of the patients needed no other form of therapy than medical therapy. This may be because most of the cases of UGIB in our cohort were severe. This assertion was substantiated by the fact that over a third of the patients presented with haemorrhagic shock. Similarly, over 80% of our patients had a Rockall score of either medium or high risk. It may also be that only severe cases were referred to the tertiary health facility for treatment, while the mild cases were treated at the peripheral centre. In addition, health care expenses are largely out-of-pocket, so this may screen out mild cases.

This study also identify that endoscopic therapy was very effective for patients who could afford it. It also significantly reduced hospital stays and the number of patients that needed surgical haemostasis. Despite fewer endoscopic options due to availability, the effectiveness of endoscopic therapy in ulcer bleeding was comparable to a previous report [42]. This study reinforces the need to improve the availability and accessibility of endoscopic therapy for patients with UGIB. This can be achieved through collaboration with world bodies such as the World Gastroenterology Organization. Involvement of non-governmental organisations in sponsoring training programs and treatment of patients with UGIB are also needed.

This study also confirmed a previous report that variceal eradication using endoscopic rubber band ligation may be more effective than injection sclerotherapy [43]. Most patients with variceal bleeding needed more than one session for eradication because of a higher grade of varices encountered in the study. Patients should be properly counselled on the need for repeated procedures with variceal eradication at the commencement of therapy to improve compliance.

Rebleeding rates in this study were very low following endoscopic therapy and were expectedly higher in patients who did not have endoscopic haemostasis. This difference was most pronounced in patients with variceal bleeding. Rebleeding is associated with increased mortality; therefore, timely identification and aggressive management of patients at high risk for continued bleeding or rebleeding have become a major focus of UGIB therapy [43].

The mortality in this study was comparable to what was reported in previous studies [26,44,45]. Most of the mortality in this study was from variceal bleeding, especially in patients who did not have endoscopic therapy. The preventable deaths of patients with cirrhosis could be avoided if treatment is made free of charge for this group of patients.

One of the challenges encountered in management of patients with UGIB in developing countries is availability of safe blood for transfusion. Blood banks are always undersupplied, meaning that patients may have to provide donors when blood is needed. Only patients with good support are able to receive enough blood during resuscitation. In fact, most of the cases of mortality arise because of a lack of donor blood. While patients with higher Rockall scores needed more blood, survival is determined by the ability to stop bleeding endoscopically or surgically.

Despite the relevance of this study to a low resource setting, we noted some inherent defects in the study. Treatments were guided by availability and affordability rather than guidelines. This reflects the current situation of most resource-poor settings. Similarly, the *H. pylori* status of the patients was determined only by histopathology of biopsy specimens rather than medical records, which were not available for analysis. This limited the interpretation of the results of *H. pylori* status may also influence rebleeding rates.

## Conclusion

In conclusion, this study clearly showed that UGIB is a common problem among Nigerians. Endoscopic therapy in a resource-poor setting like Nigeria is feasible, significantly reduces morbidity and mortality and is a cost-effective treatment. Efforts should be made to improve the accessibility of therapeutic procedures for patients with UGIB in Nigeria and other low resource settings.

## Abbreviations

UGIB: Upper gastrointestinal bleeding
GI: Gastrointestinal
*H. pylori*: *Helicobacter pylori*
NSAID: Nonsteroidal anti-inflammatory drugs
OAUTHC: Obafemi Awolowo University Teaching Hospitals Complex

## References

[1] Majno G (1975). The Healing Hand, man and Wound in the Ancient World.

[2] Esrailian E, Ian M, Gralnek IM (2005). Nonvariceal upper gastrointestinal bleeding: epidemiology and diagnosis. Gastroenterol Clin N Am.

[3] Bessa X, O’Callaghan E, Balleste B, Nieto M, Seoane A, Panades A, Vazquez DJ, Andreu M, Bory F (2006). Applicability of the Rockall score in patients undergoing endoscopic therapy for upper gastrointestinal bleeding. Dig Liver Dis.

[4] Lu Y, Loffroy R, Lau JY, Barkun A (2014). Multidisciplinary management strategies for acute non-variceal upper gastrointestinal bleeding. Br J Surg.

[5] Jensen DM, Machicado GA (2005). Endoscopic hemostasis of ulcer hemorrhage with injection, thermal, and combination methods. Tech Gastrointest Endosc.

[6] Meaden C, Makin AJ (2004). Diagnosis and treatment of patients with gastrointestinal bleeding. Curr Anaesthesia Crit Care.

[7] Sung JJ, Tsoi KK, Ma TK, Yung MY, Lau JY, Chiu PW (2010). Causes of mortality in patients with peptic ulcer bleeding: a prospective cohort study of 10,428 cases. Am J Gastroenterol.

[8] Peng Y, Chen S, Tung C, Chou W, Hu WMD, Yang S (2006). Factors associated with failure of initial endoscopic hemoclip hemostasis for upper gastrointestinal bleeding. J Clin Gastroenterol.

[9] Spiegel B, Vakil N, Ofman J (2001). Endoscopy for acute nonvariceal upper gastrointestinal tract hemorrhage: is sooner better? A systematic review. Arch Intern Med.

[10] Lee JG, Turnipseed S, Romano PS, Lee JG, Turnipseed S, Romano PS, Vigil H, Azari R, Melnikoff N, Hsu R, Kirk D, Sokolove P, Leung JW (1999). Endoscopy-based triage significantly reduces hospitalization rates and costs of treating upper GI bleeding: a randomized controlled trial. Gastrointest Endosc.

[11] Hay JA, Maldonado L, Weingarten SR, Hay JA, Maldonado L, Weingarten SR, Ellrodt AG (1997). Prospective evaluation of a clinical guideline recommending hospital length of stay in upper gastrointestinal tract hemorrhage. J Am Med Assoc.

[12] Ladep NG, Sule J, Umar SM, Obienu O, Anyanechi C, Okeke EN (2008). Oesophageal variceal band ligation using a saeed six-shooter multiband ligator, experience at Jos University Teaching Hospital, Nigeria: case report. Niger J Med.

[13] Arigbabu AO, Akinola DO (1987). Endoscopic sclerotherapy for oesophageal varices. Current status in Nigeria. Trop Doct.

[14] Forrest JA, Finlayson ND, Shearman DJ (1974). Endoscopy in gastrointestinal bleeding. Lancet.

[15] Alempijevic T, Bulat V, Djuranovic S, Kovacevic N, Jesic R, Tomic D, Krstic S, Krstic M (2007). Right liver lobe/albumin ratio: contribution to noninvasive assessment of portal hypertension. World J Gastroenterol.

[16] Rockall TA, Logan RFA, Devlin HB, Northfield TC (1996). Selection of patients for early discharge or outpatient care after acute upper gastrointestinal haemorrhage. Lancet.

[17] Cryer BL, Wilcox CM, Henk HJ, Zlateva G, Chen L, Zarotsky V (2010). The economics of upper gastrointestinal bleeding in a US managed-care setting: a retrospective, claims-based analysis. J Med Econ.

[18] Agbakwuru EA, Fatusi AO, Ndububa DA, Alatise OI, Arigbabu OA, Akinola DO (2006). Pattern and validity of clinical diagnosis of upper gastrointestinal diseases in south-west Nigeria. Afr Health Sci.

[19] Malu AO, Wali SS, Kazmi R, Macauley D, Fakunle YM (1990). Upper gastrointestinal endoscopy in Zaria, northern Nigeria. West Afr J Med.

[20] Olokoba AB, Bojuwoye BJ (2010). Indications for oesophagogastroduodenoscopy in Ilorin, Nigeria–a 30 month review. Niger J Clin Pract.

[21] Arora NK, Ganguly S, Mathur P, Ahuja A, Patwari A (2002). Upper gastrointestinal bleeding: etiology and management. Indian J Pediatr.

[22] Jaka H, Koy M, Liwa A, Kabangila R, Mirambo M, Scheppach W, Mkongo E, McHembe MD, Chalya PL (2012). A fibreoptic endoscopic study of upper gastrointestinal bleeding at Bugando Medical Centre in northwestern Tanzania: a retrospective review of 240 cases. BMC Res Notes.

[23] Zaltman C, Souza HS, Castro ME, Sobral MF, Dias PC, Lemos V (2002). Upper gastrointestinal bleeding in a Brazilian hospital: a retrospective study of endoscopic records. Arq Gastroenterol.

[24] Suba M, Mekonnen SA, Mtabho CM, Kibiki GS (2010). The aetiology, management and clinical outcome of upper gastrointestinal bleeding among patients admitted at the Kilimanjaro Christian Medical Centre in Moshi, Tanzania. Tanzania J Health Res.

[25] Mustapha S, Ajayi N, Shehu A (2009). Aetiology of upper gastrointestinal bleeding in north-eastern nigeria: a retrospective endoscopic study. The Internet Journal of Third World Medicine.

[26] Kim JJ, Sheibani S, Park S, Buxbaum J, Laine L (2014). Causes of bleeding and outcomes in patients hospitalized with upper gastrointestinal bleeding. J Clin Gastroenterol.

[27] Laine L, Yang H, Chang SC, Laine L, Yang H, Chang SC, Datto C (2012). Trends for incidence of hospitalization and death due to GI complications in the United States from 2001 to 2009. Am J Gastroenterol.

[28] Loperfido S, Baldo V, Piovesana E, Loperfido S, Baldo V, Piovesana E, Bellina L, Rossi K, Groppo M, Caroli A, Dal Bò N, Monica F, Fabris L, Salvat HH, Bassi N, Okolicsanyi L (2009). Changing trends in acute upper-GI bleeding: a population-based study. Gastrointest Endosc.

[29] van Leerdam ME (2008). Epidemiology of acute upper gastrointestinal bleeding. Best Pract Res Clin Gastroenterol.

[30] Hearnshaw SA, Logan RF, Lowe D, Hearnshaw SA, Logan RF, Lowe D, Travis SP, Murphy MF, Palmer KR (2011). Acute upper gastrointestinal bleeding in the UK: patient characteristics, diagnoses and outcomes in the 2007 UK audit. Gut.

[31] Alema ON, Martin DO, Okello TR (2012). Endoscopic findings in upper gastrointestinal bleeding patients at Lacor hospital, northern Uganda. Afr Health Sci.

[32] Gado AS, Ebeid BA, Abdelmohsen AM, Axon AT (2012). Clinical outcome of acute upper gastrointestinal hemorrhage among patients admitted to a government hospital in Egypt. Saudi J Gastroenterol.

[33] Elwakil R, Reda MA, Abdelhakam SM, Ghoraba DM, Ibrahim WA (2011). Causes and outcome of upper gastrointestinal bleeding in Emergency Endoscopy Unit of Ain Shams University Hospital. J Egypt Soc Parasitol.

[34] Ajayi AO, Adegun PT, Ajayi EA, Solomon OA, Adeoti AO, Akolawole MA (2013). Aetiology and management outcome of upper gastrointestinal bleeding in adult patients presenting at ekiti state university teaching hospital, Ado-Ekiti, Nigeria. Greener J Med Sci.

[35] Lee I, Cryer B (2011). Epidemiology and role of nonsteroidal antiinflammatory drugs in causing gastrointestinal bleeding. Gastrointest Endoscopy Clin N Am.

[36] Lanas A, Perez-Aisa MA, Feu F, Ponce J, Saperas E, Santolaria S, Rodrigo L, Balanzo J, Bajador E, Almela P, Navarro JM, Carballo F, Castro M, Quintero E, Investigators of the Asociación Española de Gastroenterología (AEG) (2005). Investigators of the Asociacio’n Espanola de Gastroenterologıca (AEG). A nationwide study of mortality associated with hospital admission due to severe gastrointestinal events and those associated with nonsteroidal anti-inflammatory drug use. Am J Gastroenterol.

[37] Hermansson M, Ekedahl A, Ranstam J, Hermansson M, Ekedahl A, Ranstam J, Zilling T (2009). Decreasing incidence of peptic ulcer complications after the introduction of the proton pump inhibitors, a study of the Swedish population from 1974–2001. BMC Gastroenterol.

[38] Sung JJ, Kuipers EJ, El-Serag HB (2009). Systematic review: the global incidence and prevalence of peptic ulcer disease [review]. Aliment Pharmacol Ther.

[39] De Berardis G, Lucisano G, D’Ettorre A, Pellegrini F, Lepore V, Tognoni G, Nicolucci A (2012). Association of aspirin use with major bleeding in patients with and without diabetes. JAMA.

[40] Kohn A, Ancona C, Belleudi V, Davoli M, Giglio L, Fusco D, Andreoli A, Perucci C, Prantera C (2010). SEID Appropriateness Working Group. The impact of endoscopy and specialist care on 30-day mortality among patients with acute non-variceal upper gastrointestinal hemorrhage: an Italian population-based study. Dig Liver Dis.

[41] Senbanjo IO, Oshikoya KA, Njokanma OF (2014). Helicobacter pylori associated with breastfeeding, nutritional status and recurrent abdominal pain in healthy Nigerian children. J Infect Dev Ctries.

[42] Kawamura T, Yasuda K, Morikawa S, Itonaga M, Nakajima M (2010). Current status of endoscopic management for nonvariceal upper gastrointestinal bleeding. Dig Endosc.

[43] Vlavianos P, Westaby D (2001). Management of acute variceal haemorrhage. Eur J Gastroenterol Hepatol.

[44] Theocharis GJ, Thomopoulos KC, Sakellaropoulos G, Katsakoulis E, Nikolopoulou V (2008). Changing trends in the epidemiology and clinical outcome of acute upper gastrointestinal bleeding in a defined geographical area in Greece. J Clin Gastroenterol.

[45] Bae S, Kim N, Kang JM, Kim DS, Kim KM, Cho YK, Kim JH, Jung SW, Shim KN (2012). Incidence and 30-day mortality of peptic ulcer bleeding in Korea. Eur J Gastroenterol Hepatol.

